# Assessment of Neuroprotective Properties of *Melissa officinalis* in Combination With Human Umbilical Cord Blood Stem Cells After Spinal Cord Injury

**DOI:** 10.1177/1759091416674833

**Published:** 2016-11-03

**Authors:** Seyed Ruhollah Hosseini, Gholamreza Kaka, Mohammad Taghi Joghataei, Mehdi Hooshmandi, Seyed Homayoon Sadraie, Kayvan Yaghoobi, Alireza Mohammadi

**Affiliations:** 1 Neuroscience Research Center, Baqiyatallah University of Medical Sciences, Tehran, Iran; 2 Department of Anatomy, School of Medicine, Iran University of Medical Sciences, Tehran, Iran; 3 Neuroscience Research Center, Shahid Beheshti University of Medical Sciences, Tehran, Iran; 4 Department of Anatomy, School of Medicine, Baqiyatallah University of Medical Sciences, Tehran, Iran

**Keywords:** astrogliosis, human umbilical cord blood, *Melissa officinalis*, myelination, spinal cord injury

## Abstract

**Introduction:**

The pathophysiology of spinal cord injury (SCI) has a classically bad prognosis. It has been demonstrated that human umbilical cord blood stem cells (hUCBSCs) and *Melissa officinalis* (MO) are useful for the prevention of neurological disease.

**Methods:**

Thirty-six adult male rats were randomly divided into intact, sham, control (SCI), MO, hUCBSC, and MO-hUCBSC groups. Intraperitoneal injection of MO (150 mg/kg) was commenced 24 hr post-SCI and continued once a day for 14 days. Intraspinal grafting of hUCBSCs was commenced immediately in the next day. The motor and sensory functions of all animals were evaluated once a week after the commencement of SCI. Electromyography (EMG) was performed in the last day in order to measure the recruitment index. Immunohistochemistry, reverse transcription-polymerase chain reaction, and transmission electron microscopy evaluations were performed to determine the level of astrogliosis and myelination.

**Results:**

The results revealed that motor function (MO-hUCBSC: 15 ± 0.3, SCI: 8.2 ± 0.37, *p* < .001), sensory function (MO-hUCBSC: 3.57 ± 0.19, SCI: 6.38 ± 0.23, *p* < .001), and EMG recruitment index (MO-hUCBSC: 3.71 ± 0.18, SCI: 1.6 ± 0.1, *p* < .001) were significantly improved in the MO-hUCBSC group compared with SCI group. Mean cavity area (MO-hUCBSC: 0.03 ± 0.03, SCI: 0.07 ± 0.004, *p* < .001) was reduced and loss of lower motor neurons (MO-hUCBSC: 7.6 ± 0.43, SCI: 3 ± 0.12, *p* < .001) and astrogliosis density (MO-hUCBSC: 3.1 ± 0.15, SCI: 6.25 ± 1.42, *p* < 0.001) in the ventral horn of spinal cord were prevented in MO-hUCBSC group compared with SCI group.

**Conclusion:**

The results revealed that the combination of MO and hUCBSCs in comparison with the control group has neuroprotective effects in SCI.

## Introduction

The spinal cord injury (SCI) is very common and has poor prognosis (Webb et al., 2010). SCI could cause severe damage to motor, sensory, and autonomic nervous system and their functions which may lead to severe disabilities (Coutts and Keirstead, 2008). The pathophysiology of SCI after the primary trauma has a vital role in the initial structural disruption. In addition, the secondary process includes the cascades of cellular and biological disruptions which can lead to long-term spinal deficits (Dasari et al., 2014). Increased oxidative stress (Fatima et al., 2015), redox transcription factors activity as well as elevated expression of inflammatory mediators play important roles (Popovich and Jones, 2003) in the promotion of the secondary process after the initial injury. SCI results in structural deformation such as degeneration of axons, disruption of neural tissue, neural and glial cell death, and demyelination around the lesion site. Axonal regeneration is inhibited by myelin-associated inhibitors in the central nervous system (CNS; Geoffroy and Zheng, 2014) and formation of astrogliosis (Deng et al., 2011). The extent of intrinsic cell renewal alone (Li and Lepski, 2013), even after application of mitogenic agents such as variety of growth factors, is not sufficient enough for substantial recovery following SCI (Chhabra and Sarda, 2015). So, therapeutic approaches such as exogenous cell transplantation should be considered.

Human umbilical cord blood stem cells (hUCBSCs) hold great promise for therapeutic repair after SCI (Dasari et al., 2007). The human umbilical cord blood by differentiating into various neural cells can enhance motor and sensory function improvement in the animal models of SCI (Kuh et al., 2005).

Transplantation of human umbilical cord blood mononuclear cells into the injured site of spinal cord did not affect the lesion extension. The survival time of transplanted cells in the injured area was 6 weeks after treatment. The transplanted group indicated better functional recovery than the untreated ones (Rodrigues et al., 2012). There is sufficient evidence which prove that stem cell therapy could be effective in spinal cord injuries but a strategy to potentiate this stem cell transplantation results is required (Niapour et al., 2012; Chhabra and Sarda, 2015).

As there have been strong interests in finding traditional agents that may help in the prevention of inflammation and disruption of neural tissues in CNS, a well-known herbal drug which has exhibited antioxidant and neuroprotective effects is *Melissa officinalis* (MO). MO is commonly known as lemon balm (family: Lamiaceae); it is one of the oldest and still the most common medicinal plants (Kamdem et al., 2013). It has been reported that the most commonly known therapeutic properties of MO extract were sedative, antispasmodic, carminative, antibacterial, antiviral, antiinﬂammatory, antioxidant, as well as neuroprotective (Kamdem et al., 2013). It was previously shown that the effective dose of MO was 150 mg/kg in SCI contusive model, and it was also shown that MO extract was effective in improving motor, sensory, and cellular function after injury (Hosseini et al., 2015). Therefore, the aim of this study is to assess the effectiveness of the MO extract in combination with hUCBSC transplantation after contusive SCI in Wistar rats. It was hypothesized that the combination of MO and hUCBSCs may play a role in preventing harmful effects and neural damage triggered by SCI.

## Materials and Methods

### Animals

After obtaining the approval of the Institutional Review Board of our university, all experiments were carried out in accordance with the Guidelines for the Animal Care and use ethics committee of the Baqiyatallah University of medical sciences. Thirty-six adult male Wistar rats weighing from 190 to 220 g were maintained under standard laboratory conditions. Animals were housed in an environment of 21 ± 2℃ with a relative humidity of 50 to 10% and a 12-hr light–dark cycle. Food and water were always available.

### Surgical Procedure for SCI

To make SCI, the animals were anesthetized with 80 mg/kg ketamine hydrochloride and 10 mg/kg xylazine hydrochloride (Alfasan Company, Netherlands) intraperitoneally. Weight-drop contusion method was conducted to induce SCI in rats. The skin and subcutaneous tissues in the thoracolumbar T12-L1 region were incised. After penetration of paravertebral muscle fascia, muscles were peeled laterally using blunt dissection forceps. The spinal cord segment at T12-L1 level was exposed by total laminectomy. The animals were subjected to an impact of 10 g weight (stainless steel rod, 3 mm diameter tip) dropped vertically in the center of the exposed spinal cord from the height of 25 mm (severe; Agrawal et al., 2010). In the sham group, all mentioned procedures were carried out, except the spinal cord contusion. The final procedure was incision suturing (Byrnes et al., 2010).

Core body temperature of animals was maintained at 36.5 to 37.5℃ during and after the study procedures. The rats were treated with gentamicin (40 mg/kg, intramuscular injection; Caspian Tamin Company, Iran) twice a day for the first 3 days as prophylaxis against urinary tract infection. The urinary bladders were pressed three times a day by the time that bladder function returned to normal. The rats were also injected subcutaneously with 25 ml/kg lactated Ringer’s solution (Caspian Tamin Company, Iran) for 2 days after SCI as once a day (Edalat et al., 2013).

### Plant Collection and Extractions

The plant was taken from the commercial source. The dried leaves powder of MO was macerated at room temperature in 70% ethanol (1 g/10 ml) and extracted for a week. On the seventh day, the ethanolic extract was refined and the extract was evaporated under reduced pressure to remove the ethanol. The dry extract was suspended in the normal saline and thus alcoholic extract of MO was prepared. We have previously shown that most effective dose of MO in the animal model of SCI is 150 mg/kg. So, in this study, we administered 150 mg/kg of MO (Pereira et al., 2009; Hosseini et al., 2015).

### Animal Groups and Drugs Administration

Rats were randomly divided into six groups as follows: Group І: intact group (*n* = 5), Group ІІ: sham rats were subjected to laminectomy without SCI (*n* = 5), Group ІІІ: rats were subjected to laminectomy and SCI (*n* = 5), Group ІV: rats were subjected to laminectomy, SCI, and treated with 150 mg/kg MO (SCI-MO; *n* = 7), Group V: rats were subjected to laminectomy, SCI, and treated with hUCBSCs (SCI-hUCBSC; (*n* = 7), Group VІ: rats were subjected to laminectomy, SCI, and treated with combination of 150 mg/kg MO and hUCBSCs (SCI-MO-hUCBSC; *n* = 7). hUCBSCs were transplanted intraspinally 24 hr after injury. MO was daily injected intraperitoneally into treatment rat groups starting 1 day after injury for 14 days.

### Culture of hUCBSCs

Human umbilical cord blood was collected from healthy women aged between 20 and 40 years with informed consent and according to the protocol approved by Institutional Review Board of Baqiyatallah University of Medical Sciences. Thereafter, human umbilical cord blood was transferred into Falcon tube containing phosphate buffered saline (PBS) without Ca^2+^ or Mg^2+^ supplemented with 2.5 µg/ml fungizone, 100 µg/ml streptomycin, 100 U/ml penicillin, and 0.5 Mm ethylenediaminetetraacetic acid (All from Merck, Germany). Mononuclear cells were separated utilizing Ficoll-Hypaque (Sigma, St. Louis, MO) density gradient centrifugation and washed out in PBS. Thereafter, the cell pellet in the tube was suspended in Dulbecco's modified Eagle medium and Ham’s F-12 (DMEM-F12) medium supplemented with 10% fetal bovine serum (FBS; All from Sigma) and cultured in tissue culture plates. The cells were kept in the 37℃ incubator with 5% CO_2_ and saturated humidity. After removing nonadherent cells in the second day of incubation, the culture of adherent cells continued until 70% confluency (Dasari et al., 2007; Faramarzi et al., 2016). At Passage 4, the cells were checked for the properties of mesenchymal stem cells using fibronectin (+), CD44 (+), and CD45 (−) (Santa Cruz Biotechnology, Santa Cruz, CA) immunostaining. The dissociated mesenchymal cells were further dispersed in 10% FBS-DMEM and counted under a microscope with the aid of a hemocytometer. The mesenchymal cells were then utilized directly for cultures or stored in liquid nitrogen for later use.

### 5-Bromo-2′-Deoxyuridine Labeling

To enable the visualization of hUCBSCs after their transplantation into the spinal cord, their nuclei were labeled with 5-bromo-2′-deoxyuridine (BrdU). BrdU (Merck, Germany) at the final concentration of 10 mg/ml was added to the culture of the hUCBSCs 24 hr before transplantation. The excess tracer was washed out with PBS and cells were suspended in fresh culture medium to obtain approximately 300,000 cells in 10 μl.

### Intra-Spinal Grafting of hUCBSCs

Animals were reanesthetized as described earlier, and the laminectomy site was re-exposed. Sham group animals were injected 24 hr after laminectomy with 9 µl of normal saline by utilizing a10 µl Hamilton syringe (Sigma). The hUCBSCs-treated group was injected 24 hr after injury. The mononuclear cell layer of hUCBSC (3 × 10^5^ ells/µL) in 9 µl of normal saline at a rate of 0.25 µl/min was transplanted into the three sites of lesion area (epicenter, distal, and proximal) at a depth of 1.2 mm. The hUCBSCs were previously labeled with BrdU so as to facilitate the identification of the cells within the subsequent histological specimens. Cyclosporine A (10 mg/kg; (Sigma) was administered as an immunosuppressant for 7 days after transplantation of hUCBSCs (Dasari et al., 2007).

### Neurological Examination

For assessment of neurological function, the Basso–Beattie–Bresnahan (BBB) scale was used for open field motor testing in all rat groups. The BBB scale is a 21-point scale ranging from 0 to 21 (Barros Filho and Molina, 2008), rating locomotion on aspects of hind limb function such as weight support, stepping ability, coordination, and toe clearance (Byrnes et al., 2010). All functional scores were obtained on Days 1, 7, 14, 21, 28, 35, 42, 49, and 56 by two individuals who were blinded to treatment. The final score of each animal was the mean value of both examiners (Hosseini et al., 2015).

Behavioral test for evaluation of sense of pain was performed by means of hot water test for the hind limbs after SCI (scores were obtained on Days 1, 7, 14, 21, 28, 35, 42, 49, and 56). The response to heat stimulation was measured by the latency of hind limb paw withdrawal of hot water at 50℃. Both paws of rats were kept in a hot water container, respectively. For each rat, six trials were obtained (three trials for each paw), and mean of this trials were recorded, and nonresponders were removed from the hot water container after 60 s (Kim et al., 2013; Hosseini et al., 2015).

### Electrophysiological Evaluations

Spontaneous rest activity was recorded from hind limb flexor muscle bilaterally. Electromyography (EMG) recording was done by 23 gauge needles for 10 s one day prior to sacrifice of animals. The EMG signal was amplified (Grass, Astro-Med, West Warwick, RI), digitized (5 kHz, Digi-data 1322A; Axon instruments, Foster City, CA), and filtered (30–300 Hz; Fouad et al., 2013). After recording, the recruitment index of motor units was acquired via compression of 10 s of recording to 1 s by EMG software. The recruitment index was scored on an ordinal scale (0 to ++++) (Stålberg et al., 1998; Hosseini et al., 2015).

### Histology and Immunohistochemistry

On Day 57, all rats were anesthetized (100 mg/kg sodium pentobarbital, I.P.). Thereafter intracardially perfused with 0.9% saline followed by 10% buffered formalin. A spinal cord segment at the level of T12-L1 was dissected, postfixed in 10% buffered formalin overnight, cryoprotected in 30% sucrose for 48 hr and serially transverse sectioned using a cryostat (B1155800 Sakura) at 10 µm thickness. All sections were processed for hematoxylin and eosin staining and assessed under light microscopy (Byrnes et al., 2010). Standard immunohistochemistry for the glial scar (glial fibrillary acidic protein) and myelination (myelin basic protein [MBP]) was performed for all of the sections. For immunohistochemistry, sections from formalin-fixed, paraffin-embedded spinal cord tissues were dewaxed, rehydrated, and retrieval of antigens was performed. After incubation with 3% H_2_O_2_ in methanol, and then normal nonimmune goat serum, the sections were incubated with rabbit antiactive glial fibrillary acidic protein (GFAP) polyclonal antibody and mouse monoclonal MBP primary antibody (Santa Cruz Biotechnology), at a dilution of 1:200 at 4℃ for overnight, followed by biotinylated goat anti-rabbit IgG for 20 min at room temperature, and subsequently incubated with streptavidin–peroxidase (All from Santa Cruz Biotechnology). PBS replaced primary antibody as the negative control. 3,3′-Diaminobenzidine chromogen was applied for visualization of peroxidase activity. Finally, the sections were counterstained with hematoxylin (Fleming et al., 2006; Hosseini et al., 2015).

### Histomorphometric Analysis

The lesion area including the cavity and surrounding damaged tissue in area of 3562,500 µm^2,^ was then measured by using an image analyzing software (Motic 2.1, Italy, Cagli); in addition, the number of lower motor neurons in area of 5,700 µm^2^, the number of positive GFAP astrocyte perikaryon in ventral horn, and area of 35,625 µm^2^ were measured. Only those cells that showed clearly discernible nucleus were counted. Densities of myelin in dorsal white matter and astrogliosis in the ventral horn of spinal cord were evaluated by using of histolab software (Iran, 1392). Five sections from each case were evaluated, and mean values were obtained for each animal. Cell counting and densitometry analyzes were carried out by two observers who were blind to the specific experimental conditions of the analyzed tissues on images acquired at 40×, 400×, and 1000× magnifications(Bharne et al., 2013).

### Transmission Electron Microscopic Studies

For electron microscopy, spinal cords from five rats from each treatment group were processed into small 3 mm^3^ blocks that surrounded the injury epicenter and fixed for 1 hr in a mixture of glutaraldehyde (1.5%) and paraformaldehyde (3%), followed by washing three times in 0.1 M sodium cacodylate and 3 mM CaCl_2_. Samples were then postfixed in potassium ferrocyanide (0.8%) and osmium tetroxide (1%) for 1 hr followed by 3× washes in 0.1 M sodium cacodylate and 3 mM CaCl_2_ (All from Sigma). Ensuing a brief dH_2_O rinse, samples were embedded in Eponate 12 (Pelco, Clovis, CA) and cured at 60℃ for 2 days. Spinal cord sections (80 nm in thickness) corresponding to the site of the lesion were cut on a Reichert Ultracut E with a Diatome diamond knife, collected on formvar-coated 1 × 2 mm^2^ copper grids, and stained with uranyl acetate followed by lead citrate. Sections were examined on a Hitachi 7600 transmission electron microscope operating at 80 kV. The myelin index (MI) was measured by means of the ratio of axon diameter to axon diameter plus its myelin sheath (Wrathall et al., 1998; Pang et al., 2012).

### RNA Extraction and Reverse Transcription-Polymerase Chain Reaction

T12-L1 segments of spinal cord from various groups were homogenized and total RNAs were isolated using RNeasy Mini Kit (Qiagen, Valencia, CA) according to the manufacturer’s protocol. Approximately 1 µg of total RNA from each sample was reverse transcribed into cDNA according to the manufacturer’s instructions using the iScrip™ cDNA Synthesis Kit (Bio-Rad Laboratories, Hercules, CA). Glyceraldehyde 3-phosphate dehydrogenase (GAPDH) was applied as an internal control. We used the following sequences for the forward and reverse primers:

For MBP: Forward primer—5CTCTGGCAAGGACTCACACA3 and reverse primer—5GTCTCTTCCTCCCCAGCTA3; For GAPDH: Forward primer—5CCACCCATGGCAAATTCC3, and reverse primer—5CAGGAGGCATTGCTGATGAT3.

The housekeeping gene GAPDH was used for normalization of MBP mRNA expression. Samples were subjected to 25 to 35 cycles at 95℃ for 30 s, 60℃ for 30 s, and 72℃ for 1 min on GeneAmp PCR System 9700 (Perkin Elmer, Boston, MA) in 25 µl reaction volumes. After amplification, reverse transcription-polymerase chain reaction products were separated on a 1% agarose gel containing 0.5 mg/ml ethidium bromide. The amplified cDNA fragments were visualized under ultraviolet light (Dasari et al., 2007).

### Statistical Analyses

Data obtained from motor and sensory functions at each time point as well as electromyographic activity between different groups were analyzed utilizing two-way analysis of variance (ANOVA). The histomorphometric, immunostaining data, densitometry, and electron microscopic data were analyzed using one-way ANOVA. In both tests, ANOVA was followed by post hoc Bonferroni’s multiple comparison tests. Data were presented as the mean ± SEM and a significance level of .05 was predetermined for all statistical analysis.

## Results

In all experiments, there were no significant differences between sham and intact groups, but significant differences were seen between intact and SCI groups (*p* < .001). In fact, the main index for SCI model induction was this significance.

### Presence of fibronectin and CD44 on hUCBSCs and Tracking of Transplanted Cells in Spinal Cord Lesion Area

**Figure 1.**
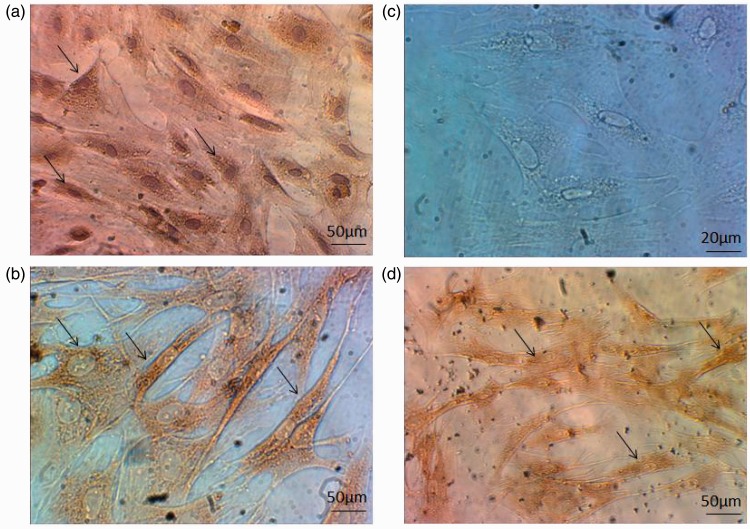
Characterization of hUCBSCs. (a) Cells stain positive for fibronectin (brown). (b) Cells stain positive (brown) for CD44. (c) Cells do not stain for CD45. (d) Cells labeled with BrdU. *Note*. hUCBSCs = human umbilical cord blood stem cells; BrdU = Br5-bromo-2′-deoxyuridine.

**Figure 2.**
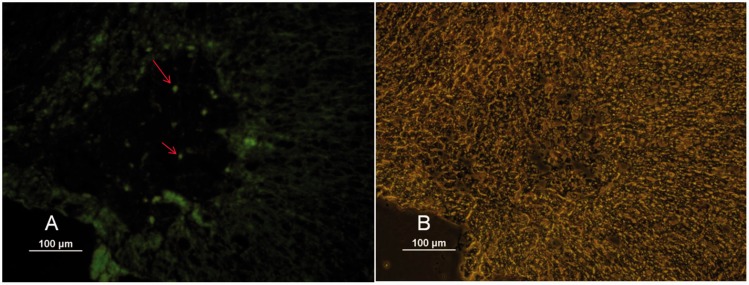
Transplanted hUCBS cells in the injured spinal cord, with anti-BrdU antibody as a primary antibody followed by the secondary antibody conjugated with FITC. The figure represents a qualitative feature of the immunostained cells (a). (b) Shows phase contrast of (a) picture. *Note*. hUCBS = human umbilical cord blood stem; BrdU = Br5-bromo-2′-deoxyuridine; FITC = fluorescein isothiocyanate.

Immunocytochemistry examination detected the localization of fibronectin and CD44 on hUCBSCs. The percentage of positivity was 89.67 and 94.78%, respectively. hUCBSCs were negative for the marker CD45 (Figure 1). Furthermore, immunofluorescence studies revealed the presence of transplanted cells in lesion area of the spinal cord (Figure 2).

### Neurological Function Results

#### Combination of MO extract and hUCBSCs transplantation increased the motor and sensory functions after SCI

**Figure 3.**
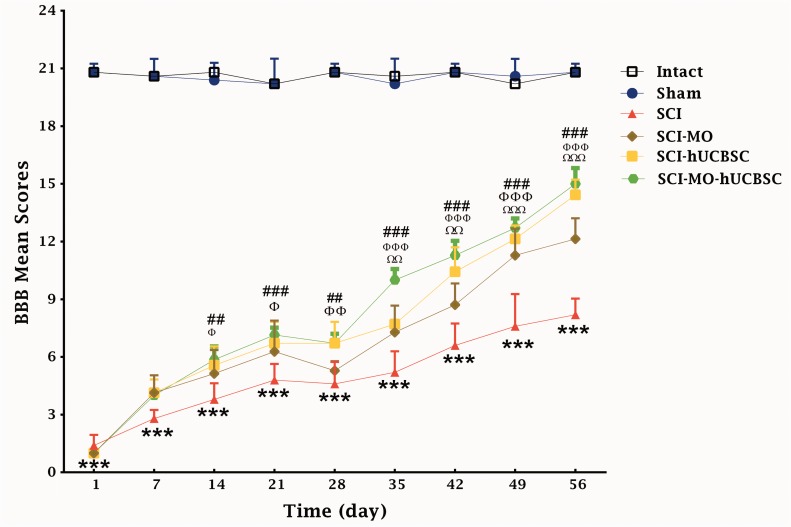
Effect of hUCBSC-MO treatment on motor function after SCI. Intraperitoneal injection of MO (150 mg/kg) was started 1 day after injury and continued once a day for 14 days after injury. Intraspinal grafting of hUCBSCs was started 24 hr after injury. Data are represented as mean of BBB score ± SEM, (*n* = 5–7) and analyzed by two-way ANOVA followed by post hoc Bonferroni’s multiple comparison test. ***p < .001 shows significant different between SCI versus intact. ΩΩ, and ΩΩΩ show significant difference between SCI-MO and SCI. Φ, ΦΦ, and ΦΦΦ show significant difference between SCI-hUCBSC and SCI. ##, and ### show significant difference between SCI-MO-hUCBSC and SCI (*p* < .05, *p* < .01, and *p* < .001, respectively). *Note*. hUCBSCs = human umbilical cord blood stem cells; MO = *Melissa officinalis*; SCI = spinal cord injury; BBB = Basso–Beattie–Bresnahan; ANOVA = analysis of variance.

SCI resulted in immediate paraplegia (loss of hind limb movement); hence the SCI group demonstrated significant changes in locomotion scores in comparison with intact group. hUCBSCs significantly enhanced locomotors function in rats when compared with SCI group. Furthermore, when intraperitoneal MO (150 mg/kg) was added a day after the injury, it significantly improved locomotors function in rats when compared with SCI group. The application of two-way ANOVA showed significant interaction between variables such as hUCBSCs therapy, MO treatment, and time, *F*(40, 270) = 29.37, *p* < .001. Application of post hoc Bonferroni’s multiple comparison tests showed significant improvement in motor function following 150 mg/kg MO treatment on Day 35, 42 (*p* < .01), 49, and 56 (*p* < .001) and hUCBSCs therapy on Day 14, 21 (*p* < .05), 28 (*p* < .01), 35, 42, 49, and 56 (*p* < .001) in comparison with SCI group. Furthermore, the combination of MO and hUCBSCs significantly enhanced motor function on Day 14, 28 (*p* < .01), 21, 35, 42, 49, and 56 (*p* < .001) in comparison with SCI group. There were no significant differences between SCI-MO, SCI-hUCBSC, and SCI-MO-hUCBSC groups (Figure 3).

**Figure 4.**
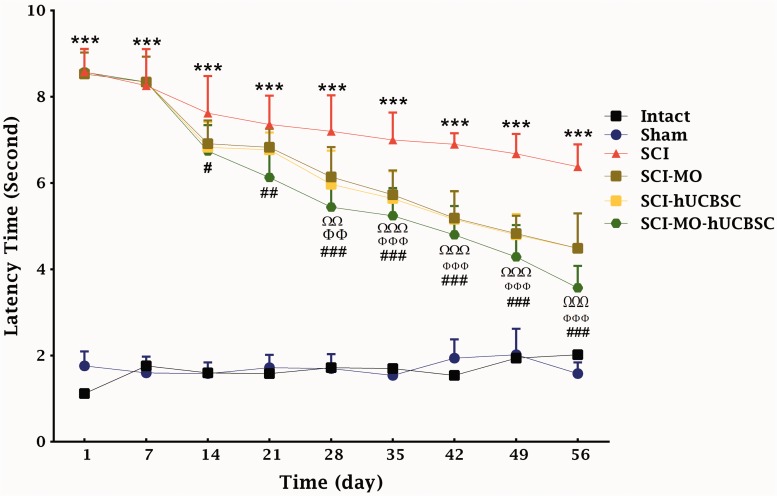
Effect of hUCBSC-MO treatment on sensory function after SCI. Intraperitoneal injection of MO (150 mg/kg) was started 1 day after injury and continued once a day for 14 days after injury. Intraspinal grafting of hUCBSCs was started 24 hr after injury. Data are represented as mean of latency time ± SEM, (*n* = 5–7) and analyzed by two-way ANOVA followed by post hoc Bonferroni’s multiple comparison test. ***p < .001 significant difference between SCI versus intact. ΩΩ, and ΩΩΩ show significant difference between SCI-MO and SCI. ΦΦ, and ΦΦΦ show significant difference between SCI-hUCBSC and SCI. #, ##, and ### show significant difference between SCI-MO-hUCBSC and SCI (*p* < .05, *p* < .01, and *p* < .001, respectively). *Note*. hUCBSCs = human umbilical cord blood stem cells; MO = *Melissa officinalis*; ANOVA = analysis of variance; SCI = spinal cord injury.

Statistical evaluations showed that the mean latency time of response to the painful stimulus decreased significantly in SCI-hUCBSC group when compared with SCI group. When intraperitoneal MO treatment (150 mg/kg) was added a day after the injury, it significantly enhanced sensory recovery in rats when compared with SCI group. The application of two-way ANOVA showed significant interaction between variables including hUCBSCs, MO treatment (150 mg/kg), and time, *F*(40, 270) = 12.39, *p* < .001. Application of post hoc Bonferroni’s multiple comparison tests showed significant improvement in sensory function following 150 mg/kg MO treatment on Day 28 (*p* < .01), 35, 42, 49, and 56 (*p* < .001) and hUCBSCs therapy on Day 28 (*p* < .01), 35, 42, 49, and 56 (*p* < .001) in comparison with SCI group. Moreover, combination of MO and hUCBSCs significantly improved motor function on Day 14 (*p* < .05), 21 (*p* < .001), 28, 35, 42, 49, and 56 (*p* < .001) in comparison with SCI group. There were no significant differences between SCI-MO, SCI-hUCBSC, and SCI-MO-hUCBSC groups (Figure 4).

### Electrophysiological Results

#### Combination of MO extract and hUCBSCs transplantation increased the recruitment pattern of hind limbs after SCI

**Figure 5.**
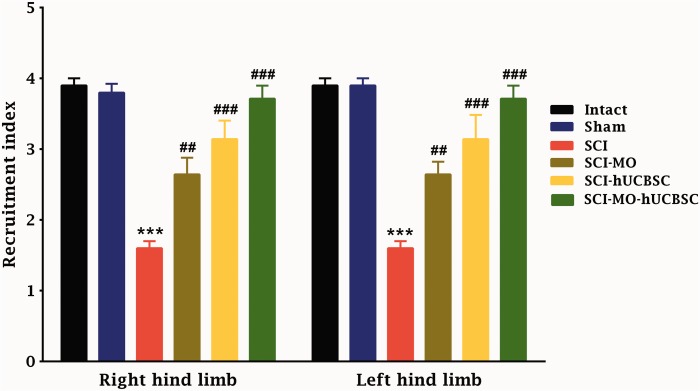
Effect of hUCBSC-MO on electromyographic activity after SCI. Intraperitoneal injection of MO (150 mg/kg) was started a day after injury and continued once a day for 14 days after injury. Intraspinal grafting of hUCBSCs was started 24 hr after injury. Data are represented as mean of recruitment index ± SEM, (*n* = 5–7) and analyzed by two-way ANOVA followed by post-hoc Bonferroni’s multiple comparison test. ***p < .001 shows significant difference between SCI versus intact. ##*p* < .01 and ###*p* < .001 versus spinal cord injury. *Note*. hUCBSCs = human umbilical cord blood stem cells; MO = *Melissa officinalis*; SCI = spinal cord injury; SEM = standard error of the mean; ANOVA = analysis of variance.

Although after the application of two-way ANOVA, there was no significant difference between the right and left hind limb. The statistical analysis indicated that the means of recruitment index were significantly increased for left and right hind limbs in SCI-MO, SCI-hUCBSC, and SCI-MO-hUCBSC groups when compared with SCI group, *F*(5, 60) = 0.01, *p* < .001. Application of post-hoc Bonferroni’s multiple comparisons test as well as Bartlett’s test for equal variances showed significant improvement in electrophysiological activity of left and right hind limbs following 150 mg/kg of MO extract administration (*p* < .01), hUCBSC therapy, and hUCBSC-MO treatment (*p* < 0.001) in comparison with SCI group. There were no significant differences between SCI-MO, SCI-hUCBSC, and SCI-MO-hUCBSC groups (Figure 5).

### Histological Results

#### Efficacy of MO-hUCBSCs treatment in reduction of cavity formation after SCI

**Figure 6.**
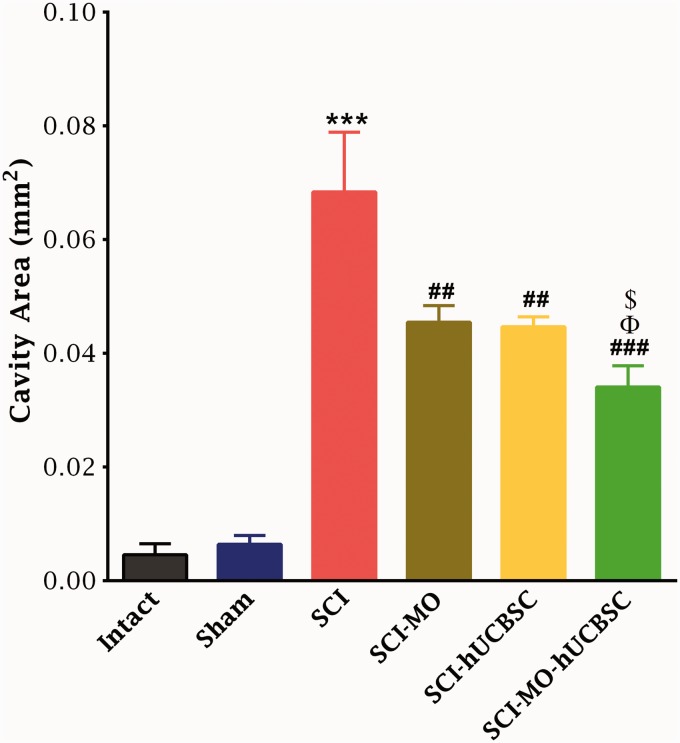
Effect of hUCBSC-MO treatment on cavity formation after SCI. Intraperitoneal injection of MO (150 mg/kg) was started one day after injury and continued once a day for 14 days after injury. Intraspinal grafting of hUCBSCs was started 24 hr after injury. Data are represented as mean of the cavity area ± SEM (*n* = 5–7) and analyzed by one-way ANOVA followed by post hoc Bonferroni’s multiple comparison test. ***p < .001 shows significant difference between SCI versus intact. ##*p* < .01, and ###*p* < .001 versus spinal cord injury. Φ shows significant difference between SCI-MO-hUCBSC and SCI-hUCBSC (*p* < .05). $ shows significant difference between SCI-MO-hUCBSC and SCI-MO (*p* < .05, *p* < .01, and *p* < .001, respectively). *Note*. hUCBSCs = human umbilical cord blood stem cells; MO = *Melissa officinalis*; SCI = spinal cord injury; SEM = standard error of the mean; ANOVA = analysis of variance.

In the intact group, spinal cord segments were not damaged in both white and gray matter. Application of one-way ANOVA demonstrated that the mean cavity size in terms of mm^2^ was significantly reduced in treatment groups, *F*(5, 30) = 27.95, *p* < .001. Moreover, post hoc Bonferroni’s multiple comparison test illustrated the significant reduction in the mean cavity area in SCI-MO, SCI-hUCBSC (*p* < .01), and SCI-MO-hUCBSC (*p* < .001) groups when compared with SCI group. Furthermore, application of one-way ANOVA showed that mean cavity area in SCI-MO-hUCBSC group was significantly reduced in comparison with SCI-MO and SCI-hUCBSC groups (*p* < .05; Figure 6).

#### Combination of MO extract and hUCBSCs transplantation decreased the lesioning of lower motor neurons in ventral horn of spinal cord after its injury

**Figure 7.**
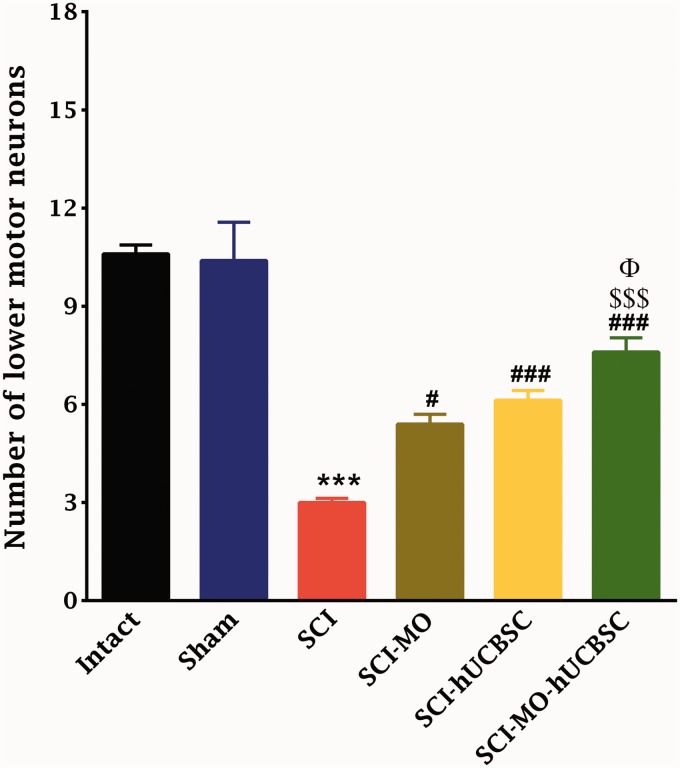
Effect of hUCBSC-MO treatment on cell loss in ventral horn of spinal cord after injury. Intraperitoneal injection of MO (150 mg/kg) was started one day after injury and continued once a day for 14 days after injury. Intraspinal grafting of hUCBSCs was started 24 hr after injury. Data are represented as mean number of ventral horn motor neurons ± SEM (*n* = 5–7) and analyzed by one-way ANOVA followed by post hoc Bonferroni’s multiple comparison test. ***p < .001 shows significant difference between SCI versus intact. #*p* < .05 and ###*p* < .001 versus spinal cord injury. Φ show significant difference between SCI-MO-hUCBSC and SCI-hUCBSC (*p* < .05). $$$ shows significant difference between SCI-MO-hUCBSC and SCI-MO (*p* < .05, *p* < .01, and *p* < .001, respectively). *Note*. hUCBSCs = human umbilical cord blood stem cells; MO = *Melissa officinalis*; SCI = spinal cord injury; SEM = standard error of the mean; ANOVA = analysis of variance.

**Figure 8.**
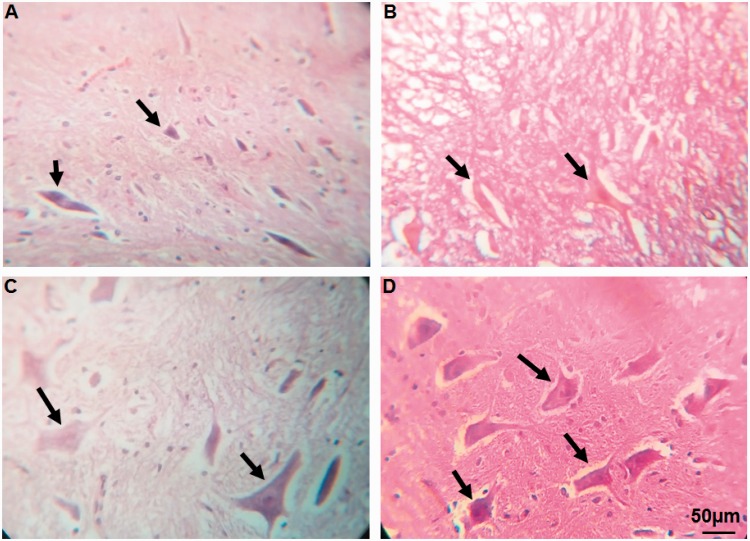
Transverse section of spinal cord showing the lower motor neurons in ventral horn of spinal cord at the level of T12-L1 of all groups which were evaluated in this study on Day 56. H&E staining showing shrinkage and reduction in ventral horn motor neurons in SCI group in comparison with SCI-MO, SCI-hUCBSC, and SCI-hUCBSC-MO groups. Black arrows illustrate the ventral horn motor neurons (400×). a = SCI, b = SCI-hUCBSC, c = SCI-MO, d = SCI-MO-hUCBSC. *Note*. H&E = hematoxylin and eosin; hUCBSC = human umbilical cord blood stem cell; MO = *Melissa officinalis*; SCI = spinal cord injury.

Statistical evaluations showed the significant differences between SCI-MO, SCI-hUCBSC, and SCI-MO-hUCBSC groups when compared with SCI group based on the number of ventral horn lower motor neurons, *F*(5, 30) = 30.86, *p* < .001. Application of post hoc Bonferroni’s multiple comparisons test as well as Bartlett’s test for equal variances revealed significant increase in the number of ventral horn motor neurons in SCI-MO (*p* < .05), SCI-hUCBSC, and SCI-MO-hUCBSC (*p* < .001) treatment groups when compared with SCI group. Moreover, application of one-way ANOVA showed significant difference in SCI-MO-hUCBSC group when compared with SCI-MO (*p* < .001) and SCI-hUCBSC (*p* < .05) groups (Figures 7 and 8).

### Immunohistochemistry and TEM Results

#### Effects of MO extract administration along with hUCBSCs transplantation on GFAP expression after SCI

**Figure 9.**
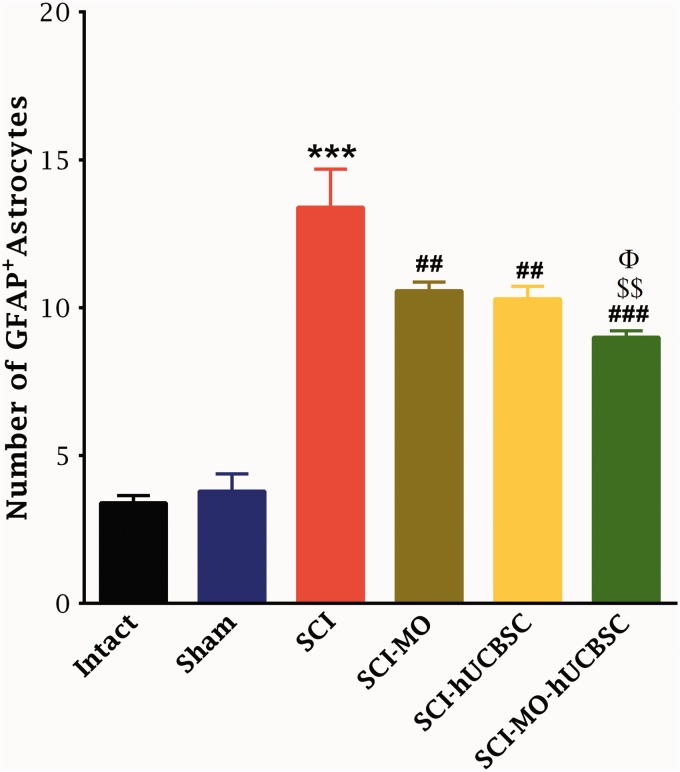
Effect of hUCBSC-MO treatment on astrogliosis formation in ventral horn of spinal cord after injury. Intraperitoneal injection of MO (150 mg/kg) was started one day after injury and continued once a day for 14 days after injury. Intraspinal grafting of hUCBSCs was started 24 hr after injury. Data are represented as mean of GFAP-positive astrocytes ± SEM (*n* = 5–7) and analyzed by one-way ANOVA followed by post hoc Bonferroni’s multiple comparison test. ***p < .001 shows significant difference between SCI versus intact. ##*p* < .01, and ###*p* < .001 versus spinal cord injury. Φ shows significant difference between SCI-MO-hUCBSC and SCI-hUCBSC (*p* < .05). $$ shows significant difference between SCI-MO-hUCBSC and SCI-MO (*p* < .05, *p* < .01, and *p* < .001, respectively). *Note*. hUCBSCs = human umbilical cord blood stem cells; GFAP = glial fibrillary acidic protein; MO = *Melissa officinalis*; SCI = spinal cord injury; SEM = standard error of the mean; ANOVA = analysis of variance.

**Figure 10.**
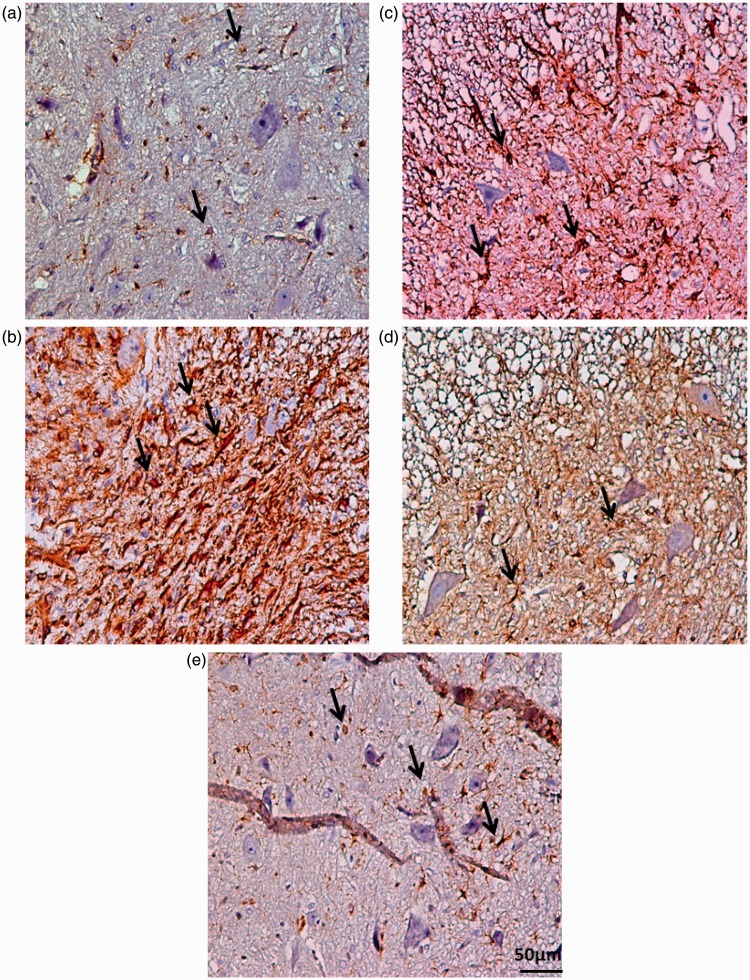
Transverse section of spinal cord showing the ventral horn gray matter of spinal cord at the level of T12-L1 of all groups which were evaluated in this study on Day 56. Black arrows illustrate the GFAP astrocytes. Reduced GFAP astrocytes are evident. a = Intact, b = SCI, c = SCI-MO, d = SCI-hUCBSC, e = SCI-MO-hUCBSC. Bar = 50 µm. (ECLIPSE 5Oi microscope). *Note*. GFAP = glial fibrillary acidic protein; hUCBSC = human umbilical cord blood stem cell; MO = *Melissa officinalis*; SCI = spinal cord injury.

Statistical evaluations revealed that the number of GFAP^+^ astrocytes was significantly increased in SCI group. However, this activation was significantly attenuated in the treatment groups, *F*(5, 30) = 45.49, *p* < .001. Application of post hoc Bonferroni’s multiple comparisons test as well as Bartlett’s test for equal variances showed a significant reduction in the GFAP expression in SCI-MO, SCI-hUCBSC (*p* < .01), and SCI-MO-hUCBSC (*p* < .001) treatment groups when compared with SCI group. Statistically significant difference was found in GFAP expression between SCI-MO-hUCBSC group in comparison with SCI-MO (*p* < .01) and SCI-hUCBSC (*p* < .05) groups (Figures 9 and 10).

**Figure 11.**
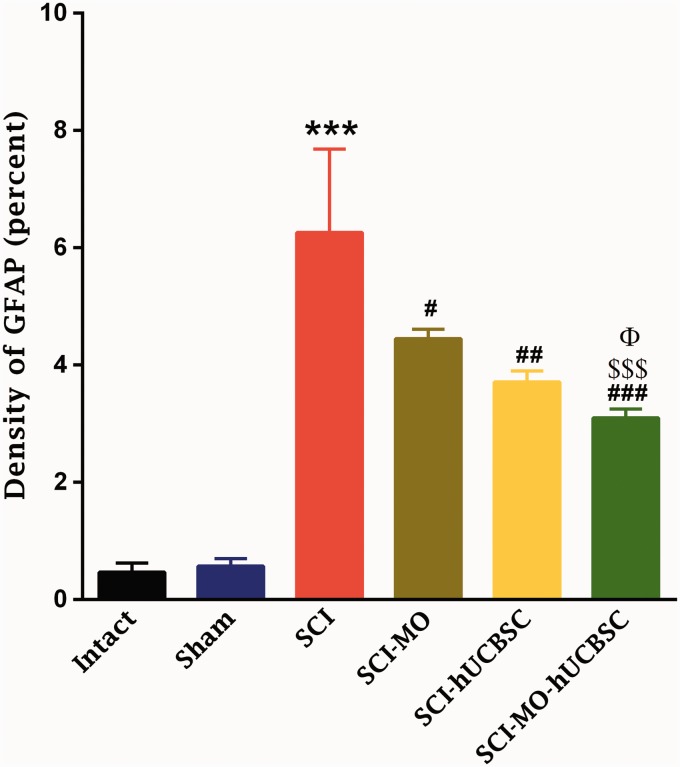
Effect of hUCBSC-MO treatment on density of astrogliosis in ventral horn of spinal cord after injury. Intraperitoneal injection of MO (150 mg/kg) was started one day after injury and continued once a day for 14 days after injury. Intraspinal grafting of hUCBSCs was started 24 hr after injury. Data are represented as mean of gliosis density ± SEM (*n* = 5–7) and analyzed by one-way ANOVA followed by post hoc Bonferroni’s multiple comparison test. ***p < .001 shows significant difference between SCI versus intact. #*p* < .05, ##*p* < .01, and ###*p* < .001 versus spinal cord injury. Φ shows significant difference between SCI-MO-hUCBSC and SCI-hUCBSC (*p* < .05). $$ shows significant difference between SCI-MO-hUCBSC and SCI-MO (*p* < .05, *p* < .01, and *p* < .001, respectively). *Note*. hUCBSCs = human umbilical cord blood stem cells; MO = *Melissa officinalis*; SCI = spinal cord injury; SEM = standard error of the mean; ANOVA = analysis of variance.

Data obtained revealed that the density of astrogliosis in the ventral horn of spinal cord was significantly reduced in treatment groups in comparison with SCI group, *F*(5, 30) = 17.66, *p* < .001. Moreover, post hoc Bonferroni’s multiple comparison test showed that the density of gliosis was significantly reduced in SCI-hUCBSC (*p* < .01) and SCI-MO-hUCBSC (*p* < .001) groups when compared with SCI group. Statistical evaluations showed significant differences between SCI-MO-hUCBSC, SCI-MO (*p* < .001), and SCI-hUCBSC (*p* < .05) groups (Figure 11).

#### Effects of MO extract administration along with hUCBSCs transplantation on myelination after SCI

**Figure 12.**
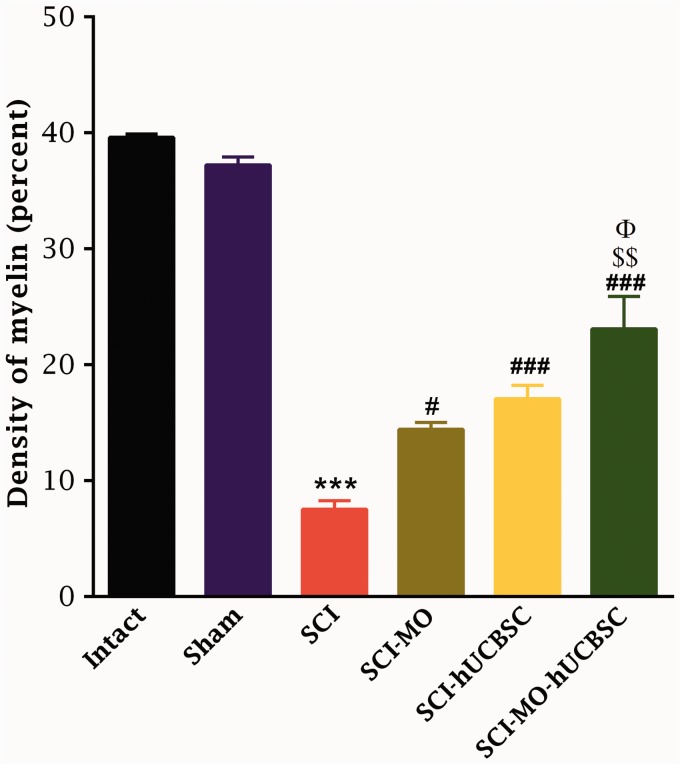
Effect of hUCBSC-MO treatment on density of myelin in dorsal white matter of spinal cord after injury. Intraperitoneal injection of MO (150 mg/kg) was started one day after injury and continued once a day for 14 days after injury. Intraspinal grafting of hUCBSCs was started 24 hr after injury. Data are represented as mean of myelin density ± SEM (*n* = 5–7) and analyzed by one-way ANOVA followed by post hoc Bonferroni’s multiple comparison test. ***p < .001 shows significant difference between SCI versus intact. #*p* < .05 and ###*p* < .001 versus spinal cord injury. Φ shows significant difference between SCI-MO-hUCBSC and SCI-hUCBSC (*p* < .05). $$ shows significant difference between SCI-MO-hUCBSC and SCI-MO (*p* < .05, *p* < .01, and *p* < .001, respectively). *Note*. hUCBSCs = human umbilical cord blood stem cells; MO = *Melissa officinalis*; SCI = spinal cord injury; SEM = standard error of the mean; ANOVA = analysis of variance.

**Figure 13.**
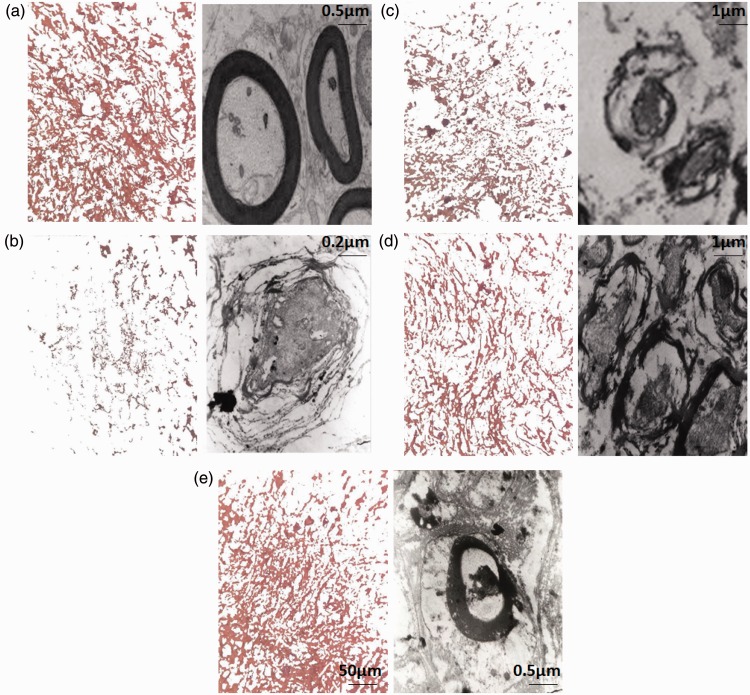
Ultra structural characteristics of myelination in dorsal white matter of spinal cord at the level of T12-L1 of all groups which were evaluated in this study on Day 56. (b) Low power view reveals the distribution of myelinated axons. Three representative high power photographs show the typical appearance of myelinated axons with extensive myelin sheath wrapped around an axon (a, d, and e). Densitometry of MBP in dorsal white matter of spinal cord at the level of T12-L1 is shown in the left part of any electron microscopy pictures. a = Intact, b = SCI, c = SCI-MO, d = SCI-hUCBSC, e = SCI-MO-hUCBSC. *Note*. MBP = myelin basic protein; hUCBSC = human umbilical cord blood stem cell; MO = *Melissa officinalis*; SCI = spinal cord injury.

Application of one-way ANOVA demonstrated that density of myelin in the dorsal white matter of spinal cord was significantly increased in treatment groups when compared with SCI group, *F*(5, 30) = 62.11, *p* < .001. Furthermore, post hoc Bonferroni’s multiple comparison test revealed that the density of myelin was significantly increased in SCI-MO (*p* < .05), SCI-hUCBSC, and SCI-MO-hUCBSC (*p* < .001) groups compared with SCI group. Furthermore, statistical evaluations showed significant differences between SCI-MO-hUCBSC, SCI-MO (*p* < .001), and SCI-hUCBSC (*p* < .05) groups (Figures 12 and 13).

**Figure 14.**
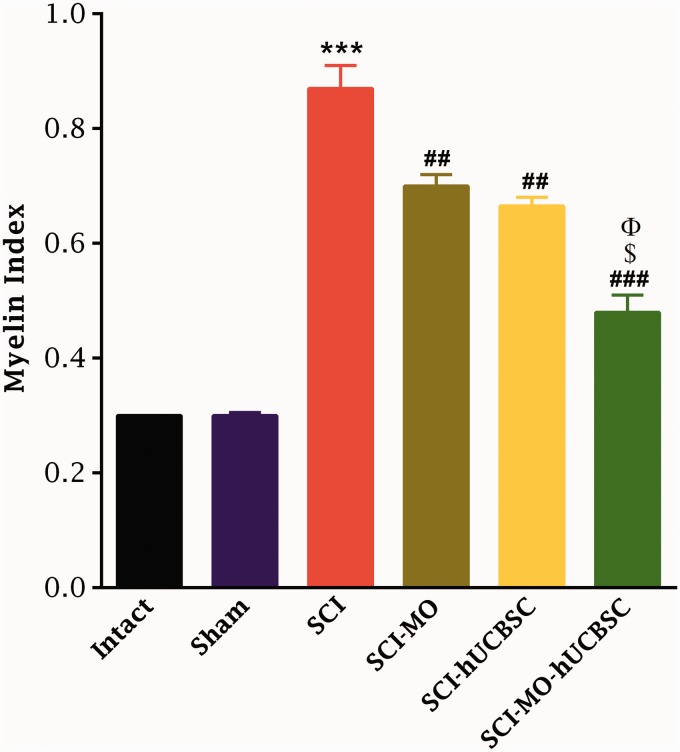
The effect of hUCBSC-MO treatment in reducing of myelin index in dorsal white matter of the spinal cord after injury. Intraperitoneal injection of MO (150 mg/kg) was started one day after injury and continued once a day for 14 days after injury. Intraspinal grafting of hUCBSCs was started 24 hr after injury. Data are represented as mean of myelin index ± SEM, (n = 2) and analyzed by one-way ANOVA followed by post-hoc Bonferroni’s multiple comparison test. ***p < .001 shows significant difference between SCI versus intact. ##*p* < .01, and ###*p* < .001 versus spinal cord injury. Φ shows significant difference between SCI-MO-hUCBSC and SCI-hUCBSC (*p* < .05). $ shows significant difference between SCI-MO-hUCBSC and SCI-MO (*p* < .05, *p* < .01, and *p* < .001, respectively). *Note*. hUCBSCs = human umbilical cord blood stem cells; MO = *Melissa officinalis*; SCI = spinal cord injury; SEM = standard error of the mean; ANOVA = analysis of variance.

Conversely, evaluation of electron microscopic pictures from all groups by application of one-way ANOVA revealed that MI was reduced in treatment groups, *F*(5, 6) = 102.1, *p* < .001. Moreover, post hoc Bonferroni’s multiple comparison test showed that MI was significantly reduced in SCI-MO, SCI-hUCBSC (*p* < .01), and SCI-MO-hUCBSC (*p* < .001) groups than in SCI group. Statistical evaluations showed significant differences between SCI-MO-hUCBSC, SCI-MO, and SCI-hUCBSC (*p* < .05) groups (Figures 13 and 14).

### RT-PCR Results

#### Combination of MO extract and hUCBSCs transplantation enhanced expression of MBP after SCI

**Figure 15.**
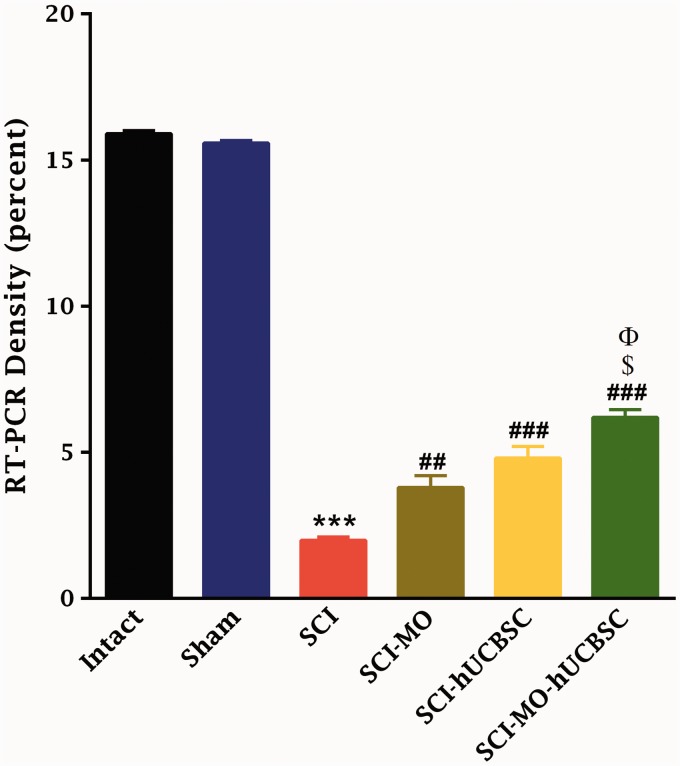
The effect of hUCBSC-MO treatment in upregulation of myelin basic protein in the spinal cord after injury. Intraperitoneal injection of MO (150 mg/kg) was started one day after injury and continued once a day for 14 days after injury. Intraspinal grafting of hUCBSCs was started 24 hr after injury. Data are represented as mean of RT-PCR bands density ± SEM (*n* = 2) and analyzed by one-way ANOVA followed by post hoc Bonferroni’s multiple comparison test. ***p < .001 shows significant difference between SCI versus intact. #*p* < .05, ##*p* < .01, and ###*p* < .001 versus spinal cord injury. Φ shows significant difference between SCI-MO-hUCBSC and SCI-hUCBSC (*p* < .05). $, $$, and $$$ show significant difference between SCI-MO-hUCBSC and SCI-MO (*p* < .05, *p* < .01, and *p* < .001, respectively). *Note*. hUCBSCs = human umbilical cord blood stem cells; MO = *Melissa officinalis*; SCI = spinal cord injury; SEM = standard error of the mean; ANOVA = analysis of variance.

**Figure 16.**
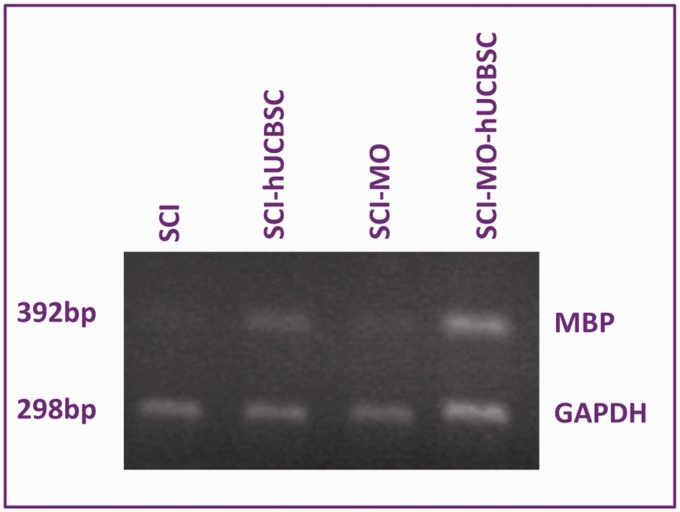
Expression of myelin basic protein in injured and treated spinal cords of rats. RT-PCR analysis of myelin basic proteins depicting SCI, SCI-MO, SCI-hUCBSC, and SCI-MO-hUCBSC groups. Housekeeping gene GAPDH was utilized as loading control. *Note*. RT-PCR = reverse transcription-polymerase chain reaction; hUCBSC = human umbilical cord blood stem cell; MO = *Melissa officinalis*; SCI = spinal cord injury; GADPH = glyceraldehyde 3-phosphate dehydrogenase.

For further confirmation of myelination process and the synthesis of MBP by MO-hUCBSC treatment after SCI, RT-PCR analysis was used. There was a change in the mRNA levels after SCI was determined utilizing standardized RT-PCR analysis. Qualitative analysis of RT-PCR findings in all groups revealed considerable upregulation of mRNA gene of MBP in SCI-MO-hUCBSC treated group when compared with SCI group. Application of one-way ANOVA revealed that the density of RT-PCR bands was increased in treatment groups, *F*(5, 6) = 1077, *p* < .001. Moreover, post hoc Bonferroni’s multiple comparison test revealed that the density of RT-PCR bands was significantly increased in SCI-MO (*p* < .01), SCI-hUCBSC. and SCI-MO-hUCBSC (*p* < .001) groups than in SCI group. Application of one-way ANOVA showed a significant difference between SCI-MO-hUCBSC, SCI-MO, and SCI-hUCBSC groups (*p* < .05; Figures 15 and 16).

## Discussion

Although some research work have indicated that stem cell transplantation for treatment of SCI is unsuccessful (Růžička et al., 2013), many investigations have demonstrated that stem cells are effective in SCI (Veeravalli et al., 2009; Caron et al., 2016; Satti et al., 2016). As a result, there has been controversy about this issue.

This research was hinged on the promotion of the therapeutic properties of hUCBSCs in combination with a neuroprotective agent to induce curative effects to SCI in rats. The present study demonstrated that combination of MO extract and hUCBSCs transplantation has neuroprotective properties in treatment of SCI. Although this combination promoted the motor, sensory, and EMG functions, there were no significant differences between SCI-MO, SCI-hUCBSCs, and SCI-MO-hUCBSC groups. A number of previous investigations have revealed functional improvement after transplantation of hUCBSCs in SCI (Saporta et al., 2003; Kuh et al., 2005; Dasari, Spomar, et al., 2008; Rodrigues et al., 2012). Previous research has shown that administration of MO extract itself improved neurological and cellular outcomes in rat SCI (Hosseini et al., 2015). MO has neuroprotective and neurotrophic effects, including promotion of functional recovery, thereby suggesting that it has therapeutic effect on neurodegenerative diseases (Bayat et al., 2012; Sepand et al., 2013). MO has acetylcholinesterase inhibitory properties (Dastmalchi et al., 2009). Anticholinesterases increase the residence time of acetylcholine in the synapse. This allows rebinding of the transmitter to nicotinic receptors. It thus gives acetylcholine the competitive advantage over the neuromuscular blocking agent (Nair and Hunter, 2004). Our results revealed that the combination of hUCBSCs and MO prevented cell loss, formation of cavity, and astrogliosis in ventral horn and also enhances the myelination in the dorsal white matter of spinal cord after injury. The effect of hUCBSCs and MO on enhancing functional recovery, myelination, and reducing astrogliosis and cavity formation is likely attributable to the inhibition of pro-inflammatory cytokines. Inflammatory processes have fundamental roles in the pathophysiology of SCI. Pro-inflammatory cytokines, including IL-1 and TNF-α, are released by activation of neurons, astrocytes, microglia, and endothelial cells after injury. Thereafter, the secondary inflammatory response and activation of IL-6 and IL-8 are induced by those cytokines (Zhu et al., 2014).

It has been shown that, transplantation of human umbilical cord blood mesenchymal stem cells (hUCB-MSCs) decreases the number of activated microglia and inhibits the permeation of immune cells and cellular apoptosis in the brain after ischemic brain injury (Yang et al., 2013; Zhu et al., 2014; Wang et al., 2016). Researchers have shown that the injection of hUCB-MSCs throughout the early stage of ischemic brain injury decreased the IL-1β, IL-6, and TNF-α expression levels in the serum and increased IL-10 expression levels. The significant increase of pro-apoptotic genes such as Bad, Bax, p53, AFAP1, caspase 3, and caspase 9 has been observed after the SCI (Sabapathy et al., 2015). IL-10 can decrease the expression of those genes, oxygen free radicals, and cytokines. Upregulation of IL-1β, IL-6, and TNF-α can initiate neuronal death and improve the synthesis of nitric oxide after injury. IL-1β can improve the intracellular calcium concentration and release neurotropic factors, which can induce neuronal apoptosis. Moreover, TNF-α can induce arachidonic acid metabolite release, which enhance extracellular accumulation of glutamate and generate neurodegenerative toxicity. Glutamate-induced cell death in the CNS contains upregulation of caspase 3 and its activation via a caspase-dependent pathway involves mitochondrial signaling. hUCBSCs decline caspase-3 and -7 activities and are responsible for activation of the Akt pathway and regulation of *N*-methyl-d-aspartic acid receptors, thereby giving neuroprotection to cortical neurons (Dasari, Veeravalli, et al., 2008). Therefore, hUCBSCs transplantation could provide neuroprotection by regulating the balance of pro- and anti-inflammatory cytokines.

Conversely, it has been shown that MO extracts have anti-inflammatory properties (Bounihi et al., 2013; Müzell et al., 2013). Its anti-inflammatory effects are due to rosmarinic acid, flavonoids, and terpenoids present in the extract. Probably, flavonoids have a more effective role by facilitating the synthesis of prostaglandin. MO administration can suppress the pro-inflammatory cytokines such as IL-1β, IL-6, and TNF-α (Bounihi et al., 2013).

Our results have demonstrated that the combination of MO extract administration and hUCBSCs transplantation prevented cell loss and enhanced myelination. A possible explanation to this is that, the transplantation of hUCBSCs can increase the length of neurofilament-positive fibers and increase the numbers of growth cone-like structures at the lesion site. Grafted hUCBSCs can survive, move over short distances, and produce large amounts of glial cell line-derived neurotrophic factors and neurotrophin-3 (NT3) in the host spinal cord (Rodrigues et al., 2012). It has also been confirmed that the hUCBSCs can form morphologically myelin sheaths in the spinal cord. It has been revealed that oligodendrocytes derived from human umbilical cord blood secrete NT3 and brain-derived neurotrophic factor. Cord blood stem cells promote the synthesis of MBP and proteolipid protein in the injured areas, thereby facilitating the process of remyelination (Dasari et al., 2009). Conversely, a large number of experimental evidences support oxidative stress as important mediators of secondary cell death after SCI (Cuzzocrea and Genovese, 2008; Jia et al., 2012; Fatima et al., 2015). It has been shown that administration of MO extract has powerful antioxidant effects which are probably exerted through the rosmarinic acid and the benzodioxole present in the extract. Moreover, compounds such as linoleic acid, carnosic acid, and ursolic acid are also present in the extracts, all of which have antioxidant properties.

The present study demonstrated that, the combination of MO and hUCBSCs inhibited astrogliosis. This issue has shown that the matrix metalloproteinase (MMP) as a proteolytic enzyme advances functional recovery after SCI by directing the development of a glial scar. Treatment with hUCBSCs after SCI altered the expression of different MMPs in rats. hUCBSCs transplantation in SCI causes upregulation of MMP-2 and therefore reduced the development of the glial scar at the lesion site (Veeravalli et al., 2009). In addition, as mentioned earlier, MO extract can inhibit the pro-inflammatory cytokines and reactive oxygen species. These two factors are key mediators of reactive astrogliosis in SCI (Bharne et al., 2013).

The limitation of this study is the nonmeasurement of the inflammatory factors and immunostaining for macrophage markers (F4/80 or Iba-1). Further investigations on differentiation of hUCBSCs along with the use of MO extracts will provide further evidence regarding the therapeutic effectiveness of hUCBSCs after SCI.

## Conclusions

SCI causes motor and sensory dysfunction, tissue deformity, and cell death, the formation of astrogliosis, and degeneration of axons. In conclusion, hUCBSCs enhanced motor and sensory dysfunction as well as promoting morphological improvement in SCI contusion model in comparison with SCI. Our results showed that MO extract can promote the neuroprotective effects of hUCBSCs. Further studies are needed to clarify the underlying mechanisms of these results.

## Author Notes

The authors alone are responsible for the content and writing of this paper.
